# Delayed Tattoo Reaction From Red Dye With Overlapping Clinicopathological Features: Examination With High-Frequency Ultrasound and Line-Field Optical Coherence Tomography

**DOI:** 10.5826/dpc.1003a53

**Published:** 2020-06-29

**Authors:** Linda Tognetti, Sean Ekinde, Cyril Habougit, Elisa Cinotti, Pietro Rubegni, Jean Luc Perrot

**Affiliations:** 1 Department of Dermatology, Division of Medical, Surgical and NeuroSciences, University of Siena, Italy; 2 Anatomopathology Service, University Hospital of Saint-Etienne, Saint-Etienne, France; 3 Department of Dermatology, University Hospital of Saint-Etienne, Saint-Etienne, France

**Keywords:** tattoo reaction, high-frequency ultrasound, line-field optical coherence tomography

## Introduction

Delayed tattoo reactions include a wide range of clinical presentations and overlapping forms; thus diagnosis can be challenging. We present a case of a delayed tattoo reaction from red dye.

## Case Presentation

**Figure 1:**
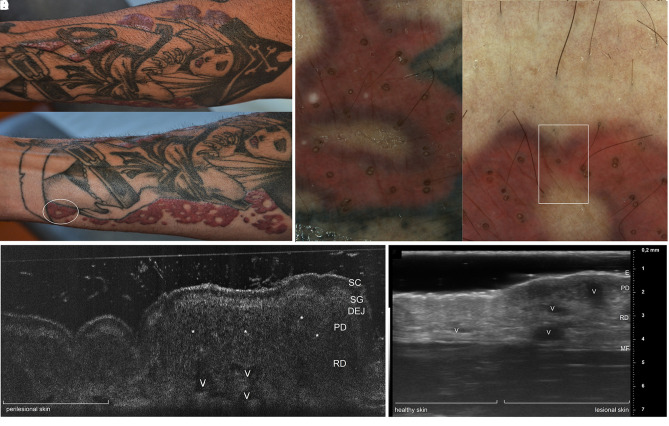
(A) Severe persistent tattoo reaction from red dye. (B) Dermoscopic examination (×20) of a swelling elevated area (white circle in A) highlights the presence of yellowish circles within the stained skin corresponding to perifollicular openings filled with keratin, dyed by exogenous pigment. Examination of transition healthy-to-lesional zone (white square) with (C) line-field confocal optical coherence tomography and (D) high-frequency ultrasound 70 MHz: inflammatory infiltrates (asterisks) and dilated vessels are visible in papillary and reticular dermis of lesional skin, generating diffuse anechoic holes in the papillary and reticular dermis, respectively. DEJ = dermoepidermal junction; E = epidermis; MF = muscularis fascia; PD = papillary dermis; RD = reticular dermis; SC = stratum corneum; SG = stratum granulosum; V = vessels.

**Figure 2:**
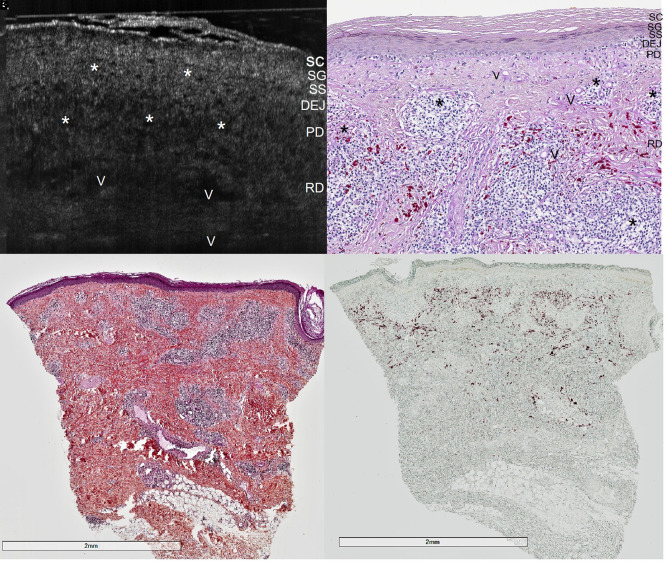
(A) Line-field confocal optical coherence tomography image (0.5 mm in depth, axial and lateral resolution of 1 μm) of lesional skin. (B) Histological correlation: PAS OM200× shows thickened and hyperreflective stratum corneum (SC) due to hyperorthokeratosis; narrowed stratum granulosum (SG) and stratum spinosum (SS); blurred dermoepidermal junction (DEJ) due to infiltrating lymphocytes; dilated vessels (V) and chronic inflammatory infiltrate (asterisks) in the papillary (PD) and reticular dermis (RD) appearing as hyporeflective areas surrounding red pigment deposits. (C) H&E staining (OM25×) demonstrates a lymphocytic infiltrate exhibiting both a lichenoid and a cluster distribution, focally periadnexal and perivascular. Red pigment deposits are distributed in the whole thickness of RD and reach the upper part of the hypodermis (HD). (D) No staining (OM25×).

An otherwise healthy 40-year-old man presented with firm elevated plaques that arose 4 months previously, within a multicolored tattoo on the right forearm, 8 months after the execution of the tattoo (Figure 1A). The patient claimed minimal and only occasional itching. Polarized dermoscopy (Luminis Visiomed, Caliber) highlighted multiple keratotic plugs within the lesion, limited to the red-dyed areas (Figure 1). Examination with line-field confocal optical coherence tomography (LC-OCT) [1] of both the transitional lesional-to-healthy part (Figure 1C) and the central part of the lesion (Figure 2A) showed a hyperkeratotic flattened epidermis and a blurred basal membrane due to the presence of inflammatory infiltrate, suggesting a lichenoid/interface dermatitis pattern. Comparative examination with high-frequency ultrasound 70 MHz (HFUS) (VEVO MD, VisualSonics) up to a depth of 5.5 mm performed at the same location revealed multiple anechoic holes and posterior shadowing due to dilated vessels, and inflammation in the superficial and deep dermis (Figure 1D). Punch biopsy taken at the same location for histopathological examination revealed hyperkeratosis, flattened epidermis with hyperkeratosis and blurring of the basal membrane; lymphocytic lichenoid inflammatory infiltrate in the superficial dermis (Figure 2B); and clustered infiltrate around red pigment deposits in reticular dermis, focally periadnexal and perivascular (Figure 2C). Immunohistochemical study revealed a mixed inflammatory chronic infiltrate composed of B and T lymphocytes. Red dye deposits were distributed in the deep dermis only and spared the superficial dermis (Figure 2D).

## Conclusions

In the last decade, delayed tattoo reactions have been on the increase due to the popularity of tattoos [2]. Metal components of the red dye (eg, nickel, mercury, and cadmium) have been addressed as the chronic antigenic stimulating agent causing a polyclonal proliferation of lymphoid cells. Traditionally, 4 histological patterns were described for delayed tattoo reactions, ie, hyperkeratotic pattern, lichenoid dermatitis, pseudolymphomatous pattern, and granulomatous pattern. However, this classification often fails to correlate significantly with clinical appearance of the lesions and has limited utility in daily practice, especially when multiple or overlapping clinicopathological features can be detected inside the same lesion [2]. Indeed, in this case we can observe keratotic cysts on dermoscopy, in favor of the hyperkeratotic pattern; histological features of lichenoid dermatitis; and evolution and clustered deep dermal infiltrate typical of the pseudolymphomatous pattern. Thus, rather than being categorized into a rigid classification, the present case should be interpreted as a severe delayed tattoo reaction from red dye with overlapping features of the pseudolymphomatous, lichenoid, and hyperkeratotic pattern.

Getting closer to an in vivo histology and providing higher resolution than conventional OCT, the new LC-OCT [1] can be proposed to better characterize the superficial part of a tattoo reaction of uncertain pattern and/or with clinical overlapping features, in combination with HFUS, which is able to reveal the inflammation in the deep dermis [1]. Moreover, the 2 techniques can be used to monitor treatment response to laser or intralesional therapy.
